# Adipose-Derived Stromal Cells and Cancer-Associated Fibroblasts: Interactions and Implications in Tumor Progression

**DOI:** 10.3390/ijms252111558

**Published:** 2024-10-28

**Authors:** Rasha S. Abo El Alaa, Wafaa Al-Mannai, Nour Darwish, Layla Al-Mansoori

**Affiliations:** Biomedical Research Centre, Qatar University, Doha P.O. Box 2713, Qatar; dr_rasha_salah2020@yahoo.com (R.S.A.E.A.); wa1901597@qu.edu.qa (W.A.-M.); nd2007863@student.qu.edu.qa (N.D.)

**Keywords:** adipose tissue, cancer, stromal cells, EMT, fibroblasts

## Abstract

Adipose-derived stromal cells (ASCs) and cancer-associated fibroblasts (CAFs) play pivotal roles in the tumor microenvironment (TME), significantly influencing cancer progression and metastasis. This review explores the plasticity of ASCs, which can transdifferentiate into CAFs under the influence of tumor-derived signals, thus enhancing their secretion of extracellular matrix components and pro-inflammatory cytokines that promote tumorigenesis. We discuss the critical process of the epithelial-to-mesenchymal transition (EMT) facilitated by ASCs and CAFs, highlighting its implications for increased invasiveness and therapeutic resistance in cancer cells. Key signaling pathways, including the transforming growth factor-β (TGF-β), Wnt/β-catenin, and Notch, are examined for their roles in regulating EMT and CAF activation. Furthermore, we address the impact of epigenetic modifications on ASC and CAF functionality, emphasizing recent advances in targeting these modifications to inhibit their pro-tumorigenic effects. This review also considers the metabolic reprogramming of ASCs and CAFs, which supports their tumor-promoting activities through enhanced glycolytic activity and lactate production. Finally, we outline potential therapeutic strategies aimed at disrupting the interactions between ASCs, CAFs, and tumor cells, including targeted inhibitors of key signaling pathways and innovative immunotherapy approaches. By understanding the complex roles of ASCs and CAFs within the TME, this review aims to identify new therapeutic opportunities that could improve patient outcomes in cancer treatment.

## 1. Introduction

**Figure 1:**
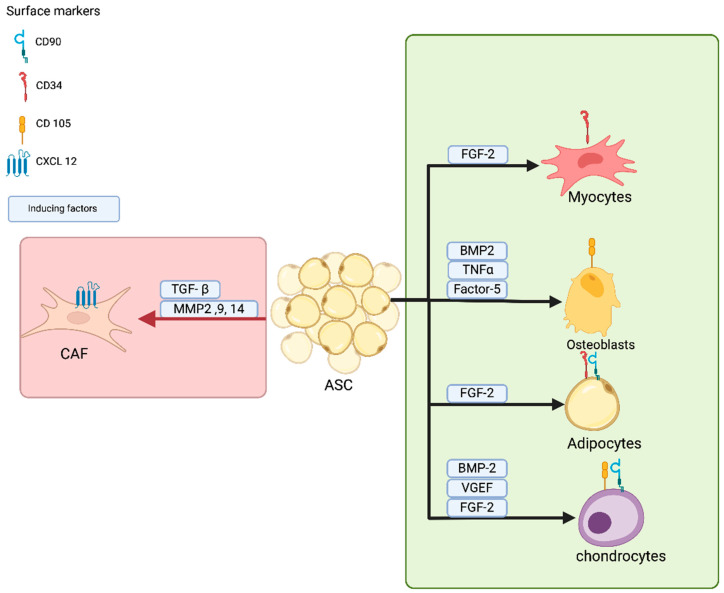
Adipose stromal cells differentiation pathways. Schematic illustration of adipose stromal cell (ASC) differentiation into various cell types, including adipocytes, osteoblasts, myocytes, and chondrocytes (green box). Key markers, such as CD34, are shown alongside factors influencing each pathway, including Epidermal growth factor2 (EGF2) for adipogenic differentiation, Bone morphogenetic protein-2 (BMP2) and Runt-related transcription factor 2 (Runx2) for osteogenic differentiation, and myoblast determination protein (MyoD) for myogenic differentiation. Tumor-derived factors induce the transformation of ASCs into CAFs (pink box). This schematic highlights the plasticity of ASCs based on microenvironmental cues.

Adipose-derived stromal cells (ASCs) are identified by specific surface markers like C luster of D ifferentiation 34, 90 and 105 (CD34, CD90, and CD105,) indicative of their mesenchymal stem cell properties [1]. Recent research has highlighted the plasticity of ASCs, demonstrating their capacity to transdifferentiate into specialized cell types under certain conditions, such as Schwann cell-like cells, showcasing their versatility and potential for regenerative applications [2]. The differentiation potential of ASCs is influenced by the surrounding microenvironment, including cytokines, growth factors, and extracellular matrix components (Figure 1) [3,4]. The tumor microenvironment (TME) significantly impacts the behavior of ASCs, promoting their differentiation into cancer-associated fibroblasts (CAFs) [5]. In cancer, the TME is characterized by a complex interplay of cellular and extracellular components that can alter the phenotype and function of resident stromal cells, including ASCs [6]. ASCs within the TME can be recruited and activated by tumor-derived signals, leading to their transformation into CAFs, which support tumor growth and progression [5]. This transformation results in further enhancement of their secretion of extracellular matrix components and pro-inflammatory cytokines, thereby promoting tumorigenesis [6]. The interaction between ASCs and cancer cells can create a feedback loop that exacerbates tumor growth, as CAFs derived from ASCs can enhance cancer cell invasiveness by remodeling the extracellular matrix and providing a supportive niche for tumor cell proliferation [7]. ASCs can also promote angiogenesis within the TME by secreting pro-angiogenic factors like Vascular endothelial growth factor (VEGF) and Interleukin 8 (IL-8), further supporting tumor growth and metastasis [5]. Moreover, ASCs can modulate the immune response within the TME, suppressing the activity of cytotoxic T cells and natural killer cells while promoting the recruitment and differentiation of immunosuppressive cell types like regulatory T cells and myeloid-derived suppressor cells [5]. This immunomodulatory function of ASCs contributes to the establishment of an immunosuppressive TME that favors tumor progression [5]. The differences between ASCs and CAFs highlight their distinct contributions to tissue homeostasis and tumor development. While ASCs play a role in maintaining tissue integrity and regeneration, their transformation into CAFs under the influence of the TME can lead to the promotion of tumor growth and metastasis [5]. This dynamic relationship underscores the role of ASCs as key players in the TME, contributing to cancer progression and potentially offering new therapeutic targets.

## 2. Epithelial-to-Mesenchymal Transition (EMT) in Cancer

**Figure 2:**
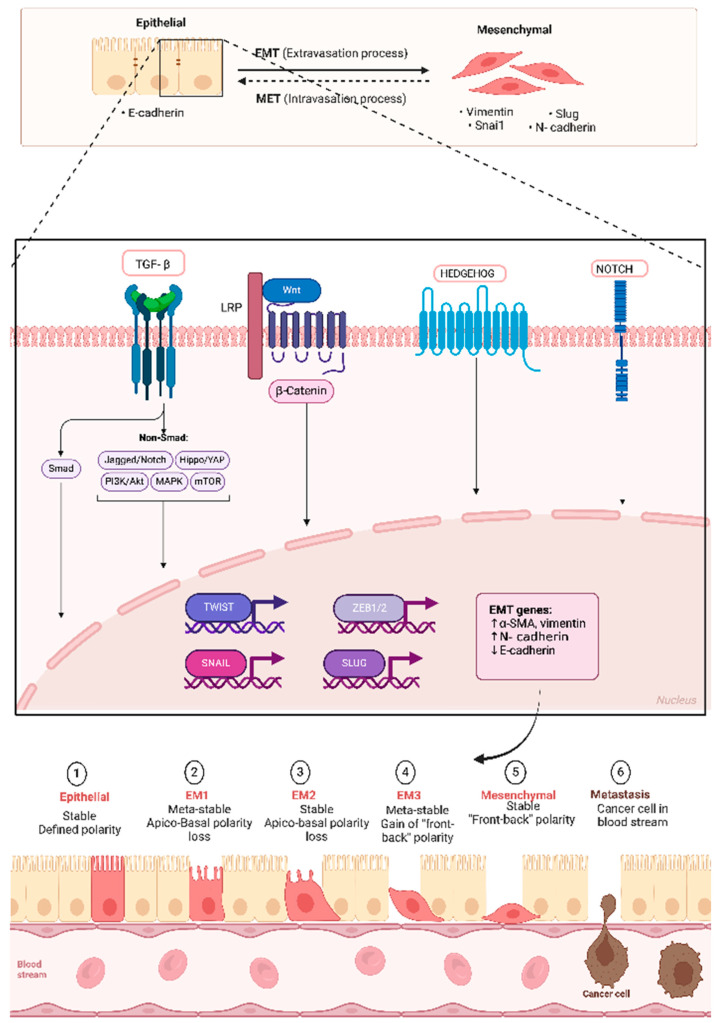
Epithelial–mesenchymal transition signaling pathways. Diagram representing key signaling pathways involved in the epithelial-to-mesenchymal transition (EMT), including TGF-β, Wnt/β-catenin, Hedgehog, and Notch pathways, and their impact on cancer cell behavior. It highlights the roles of transcription factors such as Snail, Slug, Twist, and zinc finger E-box binding homeobox 1/2 (ZEB1/2), which are activated by these pathways to repress epithelial markers and promote mesenchymal characteristics. The interplay between these signaling pathways and transcription factors underscores their critical role in facilitating the EMT, contributing to tumor progression, metastasis, and drug resistance.

The epithelial-to-mesenchymal transition (EMT) is a biological process where epithelial cells lose their cell polarity and cell–cell adhesion properties, acquiring migratory and invasive characteristics typical of mesenchymal cells [8]. This transition is crucial in various physiological processes, including embryogenesis, wound healing, and tissue regeneration, but it is also implicated in cancer progression and metastasis [9]. In cancer, the EMT facilitates the metastatic cascade by enabling tumor cells to invade surrounding tissues, enter the bloodstream, and colonize distant organs (Figure 2) [9,10]. During the EMT, cells undergo significant morphological changes, characterized by the loss of epithelial markers like E-cadherin and the gain of mesenchymal markers such as N-cadherin and vimentin (Table 1) [11]. This transition enhances cancer cells’ invasive capabilities, allowing them to penetrate the extracellular matrix (ECM) and migrate to distant sites.

Recent studies indicate that the EMT is not merely a binary switch but rather a spectrum of states, including partial EMT, where cells exhibit a hybrid phenotype with both epithelial and mesenchymal characteristics [9]. This hybrid state is often associated with increased tumor aggressiveness and the ability to evade therapeutic interventions, contributing to poor patient outcomes. The induction and regulation of the EMT are governed by a complex network of signaling pathways and transcription factors. Key molecular mechanisms are described below.

## 3. Signaling Pathways

Transforming Growth Factor-beta (TGF-β): TGF-β is one of the most potent inducers of EMT. It activates downstream signaling cascades, including Smad-dependent and non-Smad pathways, leading to the transcription of EMT-associated genes [16]. TGF-β signaling has been shown to promote not only the EMT but also therapeutic resistance, as evidenced by studies linking TGF-β-induced EMT to poor treatment outcomes in various cancers [9].

Wnt/β-catenin Signaling: This pathway plays a critical role in promoting the EMT by stabilizing β-catenin in the cytoplasm, which translocate to the nucleus and activates target genes that promote mesenchymal characteristics [17]. Recent findings suggest that Wnt signaling also interacts with metabolic pathways, further enhancing the metastatic potential of cancer cells [12].

Hedgehog and Notch Signaling: These pathways contribute to EMT induction and are involved in maintaining the stemness of cancer cells, further enhancing their metastatic potential [18]. Notably, the activation of these pathways has been associated with increased expression of immune checkpoint markers, suggesting a link between the EMT and immune evasion [9].

### 3.1. Transcription Factors

Snail, Slug, and Twist: These transcription factors are key regulators of the EMT. They repress epithelial markers and promote the expression of mesenchymal markers, facilitating the transition. For example, Snail has been shown to downregulate E-cadherin while upregulating N-cadherin, promoting cell migration and invasion [11]. Recent studies have identified copy number variations (CNVs) in these transcription factors that correlate with EMT status in various cancers, highlighting their potential as prognostic markers [19].

Zinc finger E-box binding homeobox1 and 2 (ZEB1 and ZEB2): These factors also play significant roles in the EMT by repressing epithelial characteristics and inducing mesenchymal traits. Their expression is often upregulated in various cancers and correlates with poor prognosis [10]. The regulation of ZEB proteins is complex, involving various signaling pathways and epigenetic modifications, which can further complicate their role in cancer progression [9].

### 3.2. Epigenetic Modifications

Epigenetic changes, including DNA methylation and histone modifications, contribute to the regulation of EMT-related genes. These modifications can be influenced by the tumor microenvironment and signaling pathways, leading to stable changes in gene expression that promote EMT [14]. Recent studies have shown that the tumor microenvironment can induce epigenetic reprogramming in cancer cells, facilitating the EMT process and contributing to therapeutic resistance [13].

### 3.3. Non-Coding RNAs

MicroRNAs (miRNAs) and long non-coding RNAs (lncRNAs) have emerged as important regulators of EMT. For example, miR-200 family members are known to inhibit EMT by targeting transcription factors like ZEB1 and ZEB2, thereby maintaining epithelial characteristics. Conversely, other miRNAs can promote EMT by targeting epithelial markers or enhancing the expression of mesenchymal markers [11]. Recent research has highlighted the role of lncRNAs in modulating EMT through their interactions with transcription factors and signaling pathways, further complicating the regulatory network governing this process [20].

### 3.4. ASCs Differentiation into Cancer-Associated Fibroblasts

**Figure 3:**
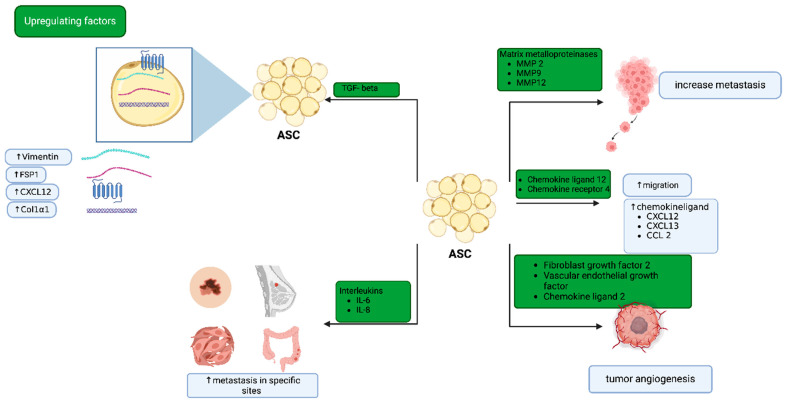
Mechanisms of the differentiation of adipose stromal cells to tumor-associated fibroblasts. This figure depicts the signaling pathways and factors (TGF-β, IL-6, Matrix Metalloproteinase (MMPs), chemokines) that induce the differentiation of adipose stromal cells (ASCs) into cancer-associated fibroblasts (CAFs). Key mechanisms include the activation of ASCs by tumor-derived factors, leading to phenotypic changes such as increased proliferation, migration, and a myofibroblast-like phenotype. CAFs contribute to tumor growth and metastasis by remodeling the extracellular matrix and secreting pro-tumorigenic factors, highlighting their role in the tumor microenvironment.

Adipose stromal cells (ASCs) can differentiate into CAFs within the tumor microenvironment, contributing to cancer progression and metastasis. Jotzu et al. demonstrated that a significant percentage of ASCs differentiate into α-smooth muscle actin (α-SMA) and tenascin-C positive CAF-like myofibroblastic phenotypes when exposed to conditioned medium from human breast cancer cell lines MDA-MB-231 and MCF7 [21]. This suggests that factors secreted by tumor cells can induce the differentiation of ASCs into CAFs [21]. Similarly, a study by Yan et al. provided evidence that cancer stem cells (CSCs) are a key source of CAFs in the tumor niche [22]. They generated CSC-like cells by treating mouse induced pluripotent stem cells with conditioned medium from breast cancer cell lines, demonstrating that these CSC-like cells formed heterogeneous populations surrounded by myofibroblast-like cells, which exhibited a CAF-like phenotype upon further analysis [22]. Several factors and signaling pathways have been implicated in the differentiation of ASCs into CAFs (Figure 3).

#### 3.4.1. Transforming Growth Factor-Beta (TGF-β)

TGF-β is a potent inducer of ASC differentiation into CAFs. Exposure to TGF-β1 can promote the expression of CAF markers such as fibroblast-specific protein 1 (FSP1), vimentin, collagen type I alpha 1 (Col1α1), and chemokine (C-X-C motif) ligand 12 (CXCL12) in ASCs [21]. TGF-β signaling not only drives the differentiation process but also enhances the tumor-promoting functions of CAFs, including their role in immune evasion and angiogenesis [5].

#### 3.4.2. Interleukins

Interleukins, particularly IL-6 and IL-8, have been shown to be upregulated in co-cultures of ASCs with various cancer cell types, including breast, colon, melanoma, and squamous cell carcinoma [5]. This upregulation is associated with increased cancer cell invasion, proliferation, and stemness [23]. IL-6, in particular, has been identified as a critical mediator in the communication between ASCs and cancer cells, promoting a pro-tumorigenic environment [24].

#### 3.4.3. Matrix Metalloproteinases (MMPs)

The co-culture of ASCs with cancer cells leads to increased expression of MMPs, such as MMP-2, MMP-9, and MMP-14, in both ASCs and cancer cells. This enhanced MMP expression is linked to increased invasion and metastasis [5,25]. MMPs facilitate the degradation of the extracellular matrix (ECM), allowing for greater tumor cell migration and invasion [5].

#### 3.4.4. Chemokines

The CXCL12/CXCR4 axis plays a role in the migration of ASCs towards the tumor site [26]. Additionally, the co-culture of ASCs with melanoma cells upregulates the expression of CXCL12/13 and CCL2, further promoting the recruitment of ASCs to the tumor microenvironment [15]. This chemokine signaling enhances the pro-tumorigenic activities of ASCs, reinforcing their role in cancer progression.

## 4. Functional Changes Induced by ASC Differentiation into CAFs

The differentiation of ASCs into CAFs results in several functional changes that affect tumor growth and metastasis (Table 2).

Tumor growth and angiogenesis: CAFs derived from ASCs secrete various growth factors and cytokines, such as fibroblast growth factor 2 (FGF2), vascular endothelial growth factor (VEGF), and CCL2, which promote both tumor growth and the formation of new blood vessels [23]. VEGF is a key pro-angiogenic factor secreted by CAFs that stimulates endothelial cell proliferation, migration, and tube formation, leading to the development of new blood vessels within the tumor [27]. Additionally, FGF2 secreted by CAFs can synergize with VEGF to enhance angiogenesis and promote tumor growth [28]. CAFs also secrete chemokines like CCL2 that recruit endothelial progenitor cells (EPCs) to the tumor site, contributing to neovascularization [29]. The recruitment of EPCs by CAF-derived CCL2 has been observed in breast cancer models, where it promotes tumor angiogenesis and growth [30]. Furthermore, CAFs can indirectly stimulate angiogenesis by remodeling the extracellular matrix (ECM) through the secretion of matrix metalloproteinases (MMPs), which release sequestered pro-angiogenic factors like VEGF [31,32].

Epithelial–mesenchymal transition (EMT): ASCs can induce the EMT in cancer cells, leading to increased invasion, metastasis, and resistance to therapy [33]. This effect has been observed in breast, lung, and glioma cancers [5]. CAFs derived from ASCs secrete factors like TGF-β, IL-6, and CXCL12 that can trigger the EMT in cancer cells, enhancing their migratory and invasive potential [11]. The induction of the EMT by CAFs also promotes the acquisition of a stem-like phenotype in cancer cells, contributing to tumor heterogeneity and therapeutic resistance [34].

Cancer stem cell maintenance: ASCs can enhance the sphere-forming capacity of breast and colon cancer cells, elevate the expression of cancer stem cell (CSC) markers, and drive tumor initiation, indicating their role in sustaining the CSC population [5,35]. CAFs derived from ASCs secrete factors like IL-6, CXCL12, and Wnt proteins that maintain the stemness of cancer cells and promote tumor-initiating potential [9]. The maintenance of CSCs by CAFs contributes to tumor heterogeneity, metastasis, and therapeutic resistance [10]. Recent studies have further elucidated the mechanisms by which ASCs and CAFs support the maintenance of CSCs; these are detailed below.

### 4.1. IL-6 Signaling

CAFs secrete high levels of IL-6, which activates the JAK/STAT3 signaling pathway in CSCs, promoting their self-renewal and tumor-initiating capacity [13]. IL-6 also induces the expression of stemness-associated genes, such as SOX2 and Nanog, in CSCs [36,37]. Blocking IL-6 signaling using neutralizing antibodies or small-molecule inhibitors can reduce CSC properties and sensitize tumors to chemotherapy [38,39].

### 4.2. CXCL12/CXCR4 Axis

CAFs secrete high levels of the chemokine CXCL12, which binds to the CXCR4 receptor expressed on CSCs [27]. This CXCL12/CXCR4 axis activates downstream signaling pathways, including PI3K/Akt and MAPK/ERK, that promote CSC self-renewal, invasion, and metastasis [40]. Disrupting this axis using CXCR4 antagonists or neutralizing antibodies can reduce CSC properties and tumor growth [40].

### 4.3. Wnt/β-Catenin Signaling

CAFs secrete Wnt ligands that activate the Wnt/β-catenin signaling pathway in CSCs [10]. This pathway is crucial for maintaining CSC properties, such as self-renewal and tumor-initiating capacity [41]. CAF-derived Wnt proteins can also induce the expression of CSC markers, such as CD44 and Epithelial cell adhesion molecule (EpCAM), in cancer cells [41]. Inhibiting Wnt/β-catenin signaling using small-molecule inhibitors or antibodies targeting Wnt ligands can reduce CSC properties and tumor growth [41].

### 4.4. Extracellular Matrix Remodeling

CAFs remodel the extracellular matrix (ECM) by secreting various matrix metalloproteinases (MMPs) and other ECM components [42]. These ECM changes can create a niche that supports CSC maintenance and tumor-initiating potential [43]. For example, CAF-derived fibronectin can activate integrin signaling in CSCs, promoting their self-renewal and tumor-initiating capacity [44].

## 5. Impact of Extracellular Vesicles (EVs) on Tumor Microenvironment Modulation

Extracellular vesicles (EVs), derived from adipose stromal cells (ASCs) and cancer-associated fibroblasts (CAFs), are pivotal in facilitating communication within the tumor microenvironment (TME) [45]. These vesicles transfer a diverse array of bioactive molecules, including proteins, lipids, and RNAs, which can profoundly impact tumor growth and immune evasion [45]. Recent research emphasizes that EVs from ASCs and CAFs are rich in microRNAs (miRNAs) and other regulatory non-coding RNAs that can alter gene expression in recipient cells [45]. For example, these EVs can convey miRNAs that induce epithelial-to-mesenchymal transition (EMT), enhancing the invasive potential of cancer cells and promoting metastasis. Additionally, proteins contained within these EVs can activate signaling pathways in tumor cells, leading to increased proliferation and survival [45].

The composition of EVs is influenced by various maternal factors, such as diet, health status, and genetic background, which further affects their roles within the TME. Studies have shown that the lipid composition of CAF-derived EVs can significantly influence their capacity to promote angiogenesis by affecting endothelial cell behavior [45]. Moreover, specific miRNAs present in these vesicles have been linked to mechanisms of immune evasion, as they can suppress the activity of cytotoxic T cells while facilitating the recruitment of immunosuppressive cell types [46].

The interaction between ASCs, CAFs, and tumor cells through EV-mediated communication establishes a dynamic feedback loop that intensifies tumor growth. By delivering signals that favor tumorigenesis, these EVs not only enhance cancer cell survival but also modify the TME to support tumor progression. Recognizing the significance of EVs in this context opens new possibilities for therapeutic strategies aimed at disrupting these communication pathways to improve treatment outcomes. Targeting the release or uptake of EVs may represent a novel approach to counteracting the pro-tumorigenic effects of ASCs and CAFs. Recent advancements in nanotechnology have led to the creation of nanoparticles designed to selectively target and neutralize EVs, potentially diminishing their impact on tumor development [47]. Furthermore, therapies aimed at altering the content of EVs or inhibiting their interactions with recipient cells could enhance the efficacy of existing treatments such as chemotherapy and immunotherapy [47,48]. These strategies underscore the potential for leveraging insights into EV-mediated communication to innovate cancer therapies.

## 6. Microbiome Influence on ASCs and CAFs

Emerging evidence suggests that the gut microbiome significantly influences adipose stromal cells (ASCs) and cancer-associated fibroblasts (CAFs), impacting their behavior within the tumor microenvironment (TME) [49]. The gut microbiota produces various metabolites, such as short-chain fatty acids (SCFAs), which can modulate the immune response and influence the differentiation and function of ASCs and CAFs [49]. For instance, SCFAs like butyrate have been shown to enhance the anti-inflammatory properties of immune cells, potentially affecting the behavior of ASCs and CAFs in a manner that could either promote or inhibit tumor progression [50].

Recent studies have highlighted that microbial metabolites can alter the secretion profiles of ASCs and CAFs, leading to changes in their interactions with tumor cells. For example, a study by Belkaid and Hand demonstrated that specific gut microbiota compositions can promote the differentiation of ASCs into CAFs by enhancing pro-inflammatory cytokine production [51]. This transformation can subsequently lead to an increased secretion of growth factors and extracellular matrix components that support tumor growth and metastasis.

Moreover, the gut microbiome may also influence the immune landscape of the TME through its effects on CAFs. Research indicates that certain microbial communities can enhance the immunosuppressive functions of CAFs, thereby facilitating tumor evasion from immune surveillance [52]. This interaction underscores the potential for targeting microbiome-derived signals as a therapeutic strategy to modulate ASC and CAF behavior in cancer.

Understanding how microbial metabolites affect ASCs and CAFs opens new avenues for therapeutic interventions aimed at manipulating these interactions to improve treatment outcomes. By harnessing the influence of the gut microbiome, it may be possible to develop strategies that either enhance anti-tumor immunity or reduce pro-tumorigenic signaling within the TME, ultimately leading to more effective cancer therapies.

## 7. Role of Metabolic Reprogramming in ASC and CAF Functionality

Metabolic reprogramming in adipose stromal cells (ASCs) and cancer-associated fibroblasts (CAFs) is a critical factor contributing to their tumor-promoting activities [53]. The tumor microenvironment (TME) induces significant metabolic changes in these cells, which alter their differentiation and secretory profiles, ultimately enhancing their support for tumor growth and progression. ASCs, when exposed to the TME, undergo metabolic shifts that favor glycolysis over oxidative phosphorylation, a phenomenon often referred to as the Warburg effect [53]. This metabolic switch enables ASCs to rapidly generate energy and biosynthetic precursors necessary for the synthesis of pro-tumorigenic factors.

These metabolic alterations not only facilitate the differentiation of ASCs into CAFs but also impact their functional roles within the TME. For instance, CAFs exhibit increased lactate production due to heightened glycolytic activity, which can create an acidic microenvironment that promotes cancer cell invasion and metastasis [53]. Additionally, altered metabolism in CAFs leads to enhanced secretion of extracellular matrix components and pro-inflammatory cytokines, further supporting tumorigenesis. The secretion of factors such as IL-6 and IL-8 by metabolically reprogrammed CAFs can activate signaling pathways in adjacent tumor cells, promoting their proliferation and survival [53].

Moreover, the metabolic plasticity of ASCs and CAFs allows them to adapt to the dynamic conditions within the TME. For example, under hypoxic conditions, these cells can switch from glycolysis to fatty acid oxidation, providing them with an alternative energy source while maintaining their tumor-supportive functions. This adaptability underscores the importance of metabolic reprogramming as a mechanism through which ASCs and CAFs contribute to tumor progression.

## 8. Clinical Implications and Therapeutic Opportunities

The transformation of adipose stromal cells (ASCs) into cancer-associated fibroblasts (CAFs) carries significant clinical implications for cancer diagnosis, prognosis, and treatment. CAFs play a crucial role in tumor progression and metastasis, and their presence can serve as a biomarker for assessing tumor aggressiveness and patient prognosis [23]. For instance, studies have shown that high levels of CAF markers correlate with poor clinical outcomes in various cancers, including breast and pancreatic cancer. Additionally, CAFs contribute to therapeutic resistance by creating a supportive environment that shields cancer cells from chemotherapy and immune attacks, complicating treatment strategies [23]. A better understanding of ASCs and their differentiated products within the tumor microenvironment could improve diagnostic methods and guide treatment decisions, particularly in personalized medicine.

### 8.1. Targeting Signaling Pathways

Given the crucial role of ASCs and CAFs in tumor promotion, various therapeutic strategies are being explored to target these cells and their interactions with the tumor microenvironment. One promising approach involves targeting key signaling pathways involved in ASC differentiation into CAFs, such as the Transforming Growth Factor-beta (TGF-β) and Platelet-Derived Growth Factor (PDGF) pathways. Inhibitors that block these pathways may reduce CAF activation and their supportive role in tumor growth [23]. For example, galunisertib, a TGF-β receptor I inhibitor, has shown potential in clinical trials by reducing CAF activity and improving patient responses to chemotherapy [54].

### 8.2. Immunotherapy Approaches

New strategies are being developed to modulate the immune response within the tumor microenvironment. Since CAFs often exhibit immunosuppressive properties, targeting the interactions between CAFs and immune cells may improve the efficacy of immunotherapies. Recent studies have suggested that CAFs can inhibit T cell infiltration and function, contributing to immune evasion [55]. Combining immune checkpoint inhibitors with therapies that disrupt CAF function could boost patient responses to treatment [23]. For instance, research has demonstrated that targeting CAFs with agents that block their immunosuppressive signals can enhance the effectiveness of Programmed Death 1/Programmed Death Ligand 1 (PD-1/PD-L1) inhibitors in preclinical models [56].

### 8.3. Exosome-Based Therapies

ASC-derived exosomes offer a novel therapeutic approach. These exosomes can be engineered to deliver anti-cancer agents or microRNAs (miRNAs) that inhibit tumor growth and metastasis [57]. For instance, exosomes loaded with miR-122 have been shown to enhance the sensitivity of hepatocellular carcinoma cells to chemotherapy, indicating that ASC-derived exosomes could serve as therapeutic vehicles in cancer treatment [23]. Moreover, ASC-derived exosomes have been found to promote the proliferation and invasion of breast cancer cells, highlighting their dual role in both tumor promotion and potential therapeutic applications [58].

### 8.4. Targeting the Tumor Microenvironment

Strategies that alter the tumor microenvironment to diminish the supportive role of CAFs are gaining momentum. Anti-angiogenic therapies that target tumor blood supply can indirectly affect CAF function by depriving them of essential growth factors [59,60]. Additionally, therapies aimed at remodeling the extracellular matrix may disrupt the supportive niche that CAFs provide for cancer cells. For instance, the use of matrix metalloproteinase (MMP) inhibitors has been explored to reduce ECM remodeling and inhibit CAF-mediated tumor progression [61].

### 8.5. Microbiom-Drevin Therapeutic Strategy

Understanding how microbial metabolites affect ASCs and CAFs opens new avenues for therapeutic interventions aimed at manipulating these interactions to improve treatment outcomes. By harnessing the influence of the gut microbiome, it may be possible to develop strategies that enhance anti-tumor immunity or reduce pro-tumorigenic signaling within the TME. For example, integrating prebiotics or probiotics with chemotherapy may not only improve patient tolerance but also potentiate therapeutic effects by modifying the TME in favor of anti-tumor immunity [62].

### 8.6. Targeting Epigenetic Modifications in ASCs and CAFs

Recent advances in understanding the role of epigenetic modifications in adipose stromal cells (ASCs) and cancer-associated fibroblasts (CAFs) have opened new avenues for therapeutic interventions aimed at inhibiting their pro-tumorigenic functions. Epigenetic changes, including DNA methylation and histone modifications, play a crucial role in regulating the expression of genes associated with the differentiation of ASCs into CAFs and their subsequent tumor-promoting activities [63]. For instance, studies have shown that the tumor microenvironment can induce specific epigenetic reprogramming in ASCs, facilitating their transformation into CAFs that support tumor growth and metastasis [64].

Targeting these epigenetic modifications presents a promising strategy to reverse the pro-tumorigenic phenotype of CAFs. Histone deacetylase (HDAC) inhibitors, for example, have been shown to alter the expression of genes involved in CAF activation and function, leading to reduced secretion of pro-inflammatory cytokines and extracellular matrix components that promote tumor progression [65]. Similarly, DNA methyltransferase inhibitors can potentially restore the expression of tumor suppressor genes silenced in CAFs, thereby inhibiting their supportive role in cancer development [66].

Moreover, the modulation of non-coding RNAs, such as microRNAs (miRNAs) and long non-coding RNAs (lncRNAs), is another promising approach to target epigenetic regulation in ASCs and CAFs. For instance, specific miRNAs have been identified that can inhibit the expression of key transcription factors involved in the epithelial-to-mesenchymal transition (EMT) process, which is crucial for CAF activation [67]. By restoring or enhancing the expression of these miRNAs, it may be possible to prevent or reverse the differentiation of ASCs into CAFs and reduce their pro-tumorigenic effects.

## 9. Fat Grafting Safety and Efficacy

Adipose tissue plays an active role in inflammation, metabolism, and tissue repair. Adipose-derived stem cells (ADSCs) promote healing by secreting growth factors and cytokines. Research shows that the extracellular fraction can enhance cell proliferation and migration without encouraging cancer growth, making it useful for post-cancer tissue repair [68,69]. Despite concerns regarding fat grafting in cancer patients, evidence indicates that ADSCs can aid in recovery from radiation-induced damage and improve the healing of wounds that do not respond to conventional therapies [70]. The regenerative properties of ADSCs include their ability to differentiate into various cell types and their paracrine effects that facilitate angiogenesis and reduce inflammation [71,72]. This dual role—both as a source of regenerative cells and as a potential risk factor for cancer recurrence—underscores the necessity for careful patient selection and further research to elucidate the mechanisms at play.

## 10. Future Directions

The roles of adipose stromal cells (ASCs) and cancer-associated fibroblasts (CAFs) in cancer progression offer both challenges and therapeutic opportunities. Future research should aim to clarify the molecular mechanisms driving ASC differentiation into CAFs and their interactions with tumor cells. Key areas of focus include understanding how gut microbiome metabolites influence ASC and CAF behavior, which could lead to dietary or probiotic interventions to modify tumor-promoting signals. Targeting signaling pathways like TGF-β and Platelet-Derived Growth Factor (PDGF) that drive ASC-CAF differentiation also hold promise, potentially enhancing current treatments by disrupting CAF support within the tumor microenvironment (TME). Exploring the role of extracellular vesicles (EVs) in cell communication could reveal new therapeutic targets by interfering with pro-tumor signaling. Moreover, integrating microbiome insights and CAF-targeting strategies into personalized medicine could improve treatment outcomes by tailoring therapies based on individual patient profiles and tumor characteristics, enhancing our ability to develop effective cancer treatments. Finally, the development of personalized medicine approaches that consider the individual tumor microenvironment may further refine therapeutic strategies and optimize patient care.

In summary, adipose stromal cells (ASCs) and cancer-associated fibroblasts (CAFs) play pivotal roles in the tumor microenvironment, significantly influencing cancer progression and metastasis. The dynamic interplay between these cells and the surrounding environment facilitates a range of tumor-promoting activities, including enhanced angiogenesis, immune evasion, and the promotion of the epithelial-to-mesenchymal transition (EMT). Recent advances in understanding the metabolic reprogramming and epigenetic modifications of ASCs and CAFs have illuminated new therapeutic opportunities aimed at disrupting their supportive roles within tumors. Targeting key signaling pathways involved in ASC differentiation into CAFs, as well as employing strategies to modulate their metabolic profiles and epigenetic landscapes, holds promise for improving treatment outcomes. Furthermore, integrating these approaches with immunotherapy could enhance anti-tumor responses by overcoming the immunosuppressive effects often exerted by CAFs. As research continues to unravel the complexities of ASCs and CAFs within the tumor microenvironment, it is essential to develop targeted therapies that not only inhibit their pro-tumorigenic functions but also leverage their potential for regenerative medicine. Ultimately, a deeper understanding of these stromal cells will contribute to more effective and personalized cancer treatment strategies, improving prognosis and quality of life for patients.

## Figures and Tables

**Table 1:** Epithelial–mesenchymal transition markers, functions, and therapeutic implications in cancer metastasis.

EMT Markers	Type	Function in EMT and Cancer Metastasis	Therapeutic Implications	References
E-cadherin	Epithelial	Maintains cell–cell adhesion; loss indicates EMT initiation and promotes metastasis	Targeting E-cadherin can restore epithelial characteristics and inhibit metastasis.	[5]
N-cadherin	Mesenchymal	Promotes cell motility and invasion; associated with aggressive tumor behavior	Inhibiting N-cadherin may reduce invasiveness and improve treatment outcomes.	[5]
Vimentin	Mesenchymal	Involved in cytoskeletal reorganization; enhances cell migration and invasion	Targeting vimentin can disrupt mesenchymal properties and reduce metastasis.	[5,12,13]
Fibronectin	Mesenchymal	Facilitates ECM remodeling; supports tumor cell migration and survival	Inhibitors of fibronectin may impair tumor progression and metastasis.	[5]
Snail	Transcription Factor	Induces EMT by repressing E-cadherin; linked to increased metastatic potential	Targeting Snail can reverse the EMT and enhance sensitivity to therapies.	[5,14]
Twist	Transcription Factor	Promotes EMT and is associated with poor prognosis in various cancers	Inhibiting Twist may restore epithelial features and reduce metastasis.	[5,14]
ZEB1	Transcription Factor	Represses E-cadherin and promotes mesenchymal traits; linked to cancer progression	Targeting ZEB1 can potentially reverse EMT and improve treatment efficacy.	[5,14]
S100A4	Mesenchymal	Promotes cell motility and invasion; associated with poor prognosis	Inhibitors targeting S100A4 may reduce metastatic potential.	[11,13,15]
FSP1	Mesenchymal	Marker for fibroblast activation; involved in EMT during fibrogenesis and cancer	Targeting FSP1 may disrupt fibroblast activation in tumors.	[12,13]
CD44	Cell Surface	Involved in cell adhesion and migration; associated with cancer stemness	Targeting CD44 may reduce tumor growth and metastasis.	[11,13,15]

**Table 2:** Comparison of adipose stromal cells (ASCs) and cancer-associated fibroblasts/tumor-associated fibroblasts (CAFs) in the tumor microenvironment.

Characteristic	Adipose Stromal Cells (ASCs)	Cancer-Associated Fibroblasts (CAFs)	Differences in Role Within TME and Impact on Cancer Progression	References
Origin	Derived from adipose tissue; multipotent stem cells	Activated fibroblasts from various origins in the tumor microenvironment	ASCs primarily aid in tissue repair, while CAFs contribute to tumor progression and metastasis.	[11,13,15]
Surface Markers	CD34, CD44, CD90, CD105, CD146	α-SMA, FAP, PDGFRα/β, S100A4, vimentin, desmin	ASCs express markers associated with stemness, while CAFs express markers linked to activation and tumor support.	[11,13,15]
Function	Tissue repair, adipogenesis, immune modulation	ECM remodeling, secretion of growth factors, immune modulation	ASCs support homeostasis and repair, whereas CAFs actively facilitate tumor growth and invasiveness.	[11,15,22]
Role in Tumor Microenvironment	Can differentiate into adipocytes or other stromal cells; may support tumor growth indirectly	Directly influence tumor cell behavior, promote angiogenesis, and create a pro-tumor environment	ASCs may have a dual role, potentially inhibiting or promoting tumors, while CAFs are predominantly tumor-promoting.	[11,15,22]
Impact on Cancer Progression	Can enhance or inhibit tumor growth depending on the context	Associated with increased tumor aggressiveness, metastasis, and poor prognosis	ASCs may limit tumor progression in some contexts, whereas CAFs are consistently linked to enhanced malignancy.	[11,13,22]
Therapeutic Implications	Potential targets for regenerative medicine and anti-cancer therapies	Targeting CAFs may improve treatment efficacy and reduce metastasis	Targeting CAFs is a promising strategy in cancer therapy, while ASCs may be leveraged for tissue regeneration.	[12,15,22]
